# Anxiety in Parkinson’s Disease Is Associated with Changes in Brain Structural Connectivity

**DOI:** 10.3233/JPD-230035

**Published:** 2023-09-08

**Authors:** Guillaume Carey, Romain Viard, Renaud Lopes, Gregory Kuchcinski, Luc Defebvre, Albert F.G. Leentjens, Kathy Dujardin

**Affiliations:** a Univ. Lille, Inserm, CHU Lille, U1172 - LilNCog - Lille Neuroscience & Cognition, Lille, France; b School for Mental Health and Neurosciences (MHeNS), Maastricht University, Maastricht, The Netherlands; c Department of Neurology and Movement Disorders, Lille University Medical Centre, Lille, France; d Univ Lille, UMS 2014 – US 41 – PLBS – Plateformes Lilloises en Biologie & Santé, Lille, France; e Department of Neuroradiology, Lille University Medical Centre, Lille, France; f Department of Psychiatry, Maastricht University Medical Centre, Maastricht, The Netherlands

**Keywords:** ClinicalTrials.gov Identifier: NCT01792843.

## Abstract

**Background:**

Anxiety in Parkinson’s disease (PD) has been associated with grey matter changes and functional changes in anxiety-related neuronal circuits. So far, no study has analyzed white matter (WM) changes in patients with PD and anxiety.

**Objective:**

The aim of this study was to identify WM changes by comparing PD patients with and without anxiety, using diffusion-tensor imaging (DTI).

**Methods:**

108 non-demented PD patients with (*n* = 31) and without (*n* = 77) anxiety as defined by their score on the Parkinson Anxiety Scale participated. DTI was used to determine the fractional anisotropy (FA) and mean diffusivity (MD) in specific tracts within anxiety-related neuronal circuits. Mean FA and MD were compared between groups and correlated with the severity of anxiety adjusted by sex, center, Hoehn & Yahr stage, levodopa equivalent daily dosage, and Hamilton depression rating scale.

**Results:**

Compared to patients without anxiety, PD patients with anxiety showed lower FA within the striato-orbitofrontal, striato-cingulate, cingulate-limbic, and caudate-thalamic tracts; higher FA within the striato-limbic and accumbens-thalamic tracts; higher MD within the striato-thalamic tract and lower MD within the striato-limbic tract.

**Conclusions:**

Anxiety in PD is associated with microstructural alterations in anxiety-related neuronal circuits within the WM. This result reinforces the view that PD-related anxiety is linked to structural alteration within the anxiety-related brain circuits.

## INTRODUCTION

Anxiety is among the most frequent neuropsychiatric symptoms in Parkinson’s disease (PD) with an average point prevalence of 31% [1]. However, the underlying mechanisms remain uncertain. Recent neuroimaging studies showed that anxiety in PD may be associated with an imbalance between two neuronal circuits [2, 3]. The fear circuit, involved in fear processing, could be over-activated in PD patients with anxiety. This circuit involves the amygdala, anterior cingulate cortex (ACC), medial prefrontal cortex (mPFC), insular cortex, hippocampus, and striatum [4–6]. In addition, the limbic cortico-striato-thalamo-cortical anxiety circuit, a dopaminergic circuit involved in the control of emotions, could be under-activated. This circuit involves the mPFC, the orbitofrontal cortex (OFC), the ACC, the ventral part of the basal ganglia (accumbens nucleus, pallidum, caudate, subthalamic nucleus) and the thalamus [7, 8]. These findings were based on MRI measures of functional connectivity and grey matter (GM) volume. White matter (WM) abnormalities have also been associated with motor symptoms and disease severity [9], cognitive decline [10], and depression [11] in PD. To date, no study has explored WM changes associated with PD-related anxiety. Diffusion tensor imaging (DTI) is a common method for exploring structural connectivity and WM changes as integrity along WM fibers through parameters such as the fractional anisotropy (FA) and the mean diffusivity (MD). These parameters are indices of axonal and myelin integrity. Changes in these parameters could thus reflect microstructural alterations in the brain [10, 11].

In PD, a recent DTI study showed that reduced FA and increased MD in fronto-occipital, insular, thalamic, and callosal regions were associated with cognitive decline [10]. A recent systematic review also reported microstructural changes (i.e., reduced FA and increased MD) in specific limbic structures such as prefrontal regions, in depressed PD patients compared to non-depressed PD patients and healthy controls [11]. In non-PD anxious patients, DTI abnormalities have been described, specifically a reduced FA in the uncinate fasciculus, a tract between limbic structures, namely the amygdala and the orbitofrontal cortex, and in the cingulum [12, 13]. These structures are part of the fear circuit.

DTI may thus help to decipher the underlying mechanisms of cognitive and behavioral symptoms in PD.

The aim of this study was to identify microstructural changes between structures involved in the fear and the limbic circuits in PD patients with anxiety compared to PD patients without anxiety, using DTI parameters such as FA and MD.

We hypothesized that FA would be reduced and MD increased in PD patients with anxiety in the anxiety-related neuronal circuits reflecting a higher level of microstructural alteration and dopaminergic neuronal degeneration.

## MATERIALS AND METHODS

### Population

One-hundred and fifty-six PD patients were enrolled from two movement disorders clinics in Lille (France) and Maastricht (The Netherlands) between March 2013 and August 2014 [14]. All the patients met the PD diagnostic criteria from the United Kingdom Parkinson’s Disease Society Brain Bank and Movement Disorders Society clinical diagnostic criteria for PD [15]. Patients with other neurological disorders or moderate to severe dementia (Movement Disorders Society criteria for Parkinson’s disease dementia [16]) were excluded.

Age, sex, duration of formal education, disease duration, history of PD or psychiatric disorders were recorded.

Non-motor symptoms, motor symptoms and disease severity were assessed using the Movement Disorder Society Unified Parkinson Disease Rating Scale (MDS-UPDRS) part I, MDS-UPDRS part IIII and Hoehn-Yahr staging [17], respectively. The levodopa equivalent daily dosages (LEDD) were calculated, and the use of antidepressant and anxiolytics treatments reported.

The Parkinson Anxiety Scale (PAS) [18], the Hamilton Depression Rating Scale (HAMD) [19], and the Lille Apathy Rating Scale (LARS) [20] were used to assess anxiety, depression, and apathy, respectively.

Vascular risk factors and cerebral WM changes (hypersignals) were assessed to control for potential confounding bias. Diabetes, hypertension, hypercholesterolemia, tobacco use, cerebral infarcts, arteriopathy, and total vascular risk factors (at least one of the reported factor) were reported. Cerebral WM changes were assessed using Fazekas scores for periventricular (P), deep (D), and total (P+D) changes. Additional information on this study group is detailed in the original paper [14]. Assessments were all performed when the patients were in the ON-drug state.

Written informed consent was obtained from all participants after full information of the procedure. The study was approved by the institutional ethics committees of both participating institutions (Lille: CPP Nord-Ouest IV, 2012-A 01317-36; Maastricht: METC AZM/UM 12-3-064).

### Characterization of anxiety

Patients were divided into two groups, one with and the other without anxiety, according to their score on the PAS, a scale specifically developed to detect anxiety in PD patients. We used the observer-rated version. Patients were considered “with anxiety” if they had a score above the defined cut-off in at least one of the three subparts of the scale (part A (persistent anxiety) >9, part B (episodic anxiety) >3, or part C (avoidance behavior) >3) [18].

### Imaging data acquisition

Patients were scanned at two sites using identical 3-Tesla Philips Achieva MRI scanner (Philips Healthcare, Best, The Netherlands) with identical software versions and MR sequences. The imaging protocol included an anatomical three-dimensional T1-weighted (3D-T1w) sequence [voxel size = 1×1×1 mm^3^, repetition time (TR) = 7.2 ms, echo time (TE) = 3.3 ms, matrix size = 256×256×176 voxels, flip angle = 9°] and a diffusion tensor imaging (DTI) sequence [voxel size = 2×2×2 mm^3^, TR = 13,000 ms, TE = 55 ms, matrix size = 128×128×66 voxels, flip angle = 90°, 64 gradient directions at b = 1,000 s/mm^2^]. To correct B0 field inhomogeneity-induced distortion, two non-diffusion-weighted images (b = 0 s/mm^2^) with opposite phase-encoding directions were also collected [21]. For quality control, all images were visually inspected by a board-certified neuroradiologist (GK).

### DTI data preprocessing

DTI data were first corrected for eddy currents and geometrical/signal distortions [22]. Eddy current artifacts were corrected using the *eddy_correct* function in the *FMRIB Software Library*. Then, the distortion field, inherent to echo planar images (EPI) in the phase encoding direction and responsible for geometric and signal artifacts, was calculated using a pair of spin echo EPI scans with opposite phase encoding directions [21]. The “*epiunwarp*” function in the *Computational Morphometry Toolkit (CMTK 3.2.25)* was used to estimate the distortion field and applied it to the DTI data.

### DTI analysis

A complete brain parcellation including 91 cortical areas and 15 subcortical areas from the FSL Harvard-Oxford Atlas was used to define regions of interest (ROI) in MNI-space [23] through *FreeSurfer* (version 5.3) [24] procedure. A non-linear registration was performed to transform these ROIs to the patient-space using the tool *ANTs* (https://github.com/ANTsX/ANTs). According to our hypotheses, five subcortical bilateral ROIs, the nucleus accumbens, amygdala, caudate, putamen, and thalamus, as well as eight cortical ROIs, the caudal ACC (cACC), rostral ACC (rACC), caudal middle frontal gyrus (cMFG), rostral middle frontal gyrus (rMFG), superior frontal gyrus (SFG), insula, lateral fronto-orbital cortex (lForb), and medial fronto-orbital cortex (mForb), were selected. The ROIs were visually inspected for each individual, and then dilated (one voxel in each direction) by masking with the lateral ventricles and brain mask.

Fiber tracking between the cortical and subcortical ROIs was performed using a probabilistic streamline tractography, as implemented in *MRtrix* software [25, 26]. Fiber pathways were generated by randomly seeding a starting subcortical ROI and tracking until the fiber reached the ending cortical or subcortical ROI to ensure symmetrical fiber tracking (maximum number of harmonics was set to 6, maximum number of fibers = 5000, FA cutoff = 0.1, curvature = 60 degrees). Fibers leaving the WM mask were terminated and discarded. Fibers obtained from the tractography solutions were then reduced to core fiber tracts by removing false positive using linear fascicle evaluation (LiFE) [27]. Next, fibers greater than 3 standard deviations away from the mean spatial position of the core fiber (Mahalanobis distance) and fibers greater or smaller than 3 standard deviations in size were removed.

Among the created tracts, those that are known to be involved in unrelated non-mental functions, such as the corticospinal tract end the longitudinal fasciculus, or those whose procedure failed were excluded from further analyses. The selected tracts are called “WM specific tracts”, in this study.

To estimate the integrity of each WM specific tract, FA and MD maps were computed for each subject using *MRTrix* process [25, 26]. FA represents a common measurement used in DTI studies ranging from 0 = isotropic movement of water molecules (e.g., cerebrospinal fluid) to 1 = anisotropic movement of water molecules (e.g., fiber tracts). It means that diffusion of molecules is allowed in only one direction. Inversely, MD, describing the average mobility of water molecules, will be higher in the cerebrospinal fluid (approx. 3×10^–3^ mm^2^/s) than in WM (approx. 5×10^–4^ mm^2^/s). The mean FA and MD values were then calculated for each patient and each tract.

### Statistical analyses

For all analyses, the statistical significance threshold was set at *p*-value <0.05. Correction for multiple comparisons (FDR – False Discovery Rate) were performed for DTI data. The normality of distribution was assessed using a Kolmogorov-Smirnov tests.

*Demographic and clinical data*. Numerical variables were described as means and standard deviations, the ordinal variables as median and range and the categorical variables as frequencies and percentages.

Qualitative data were compared using Odds Ratio’s and quantitative data using two sample T-tests or Mann-Whitney tests depending on normality of the distribution. These analyses were performed with SPSS-IBM, version 26 (SPSS, Chicago).

*Comparison analyses*. The mean FA and MD values of each specific tract were compared between the two groups using an ANCOVA procedure with center, sex, Hoehn-Yahr stage, LEDD and HAMD score as covariates. We ensured that all comparisons met the assumptions of ANCOVA procedure.

*Regression analyses*. Hierarchical multiple regression analyses were performed to examine the relationship between the PAS score and the mean FA and MD values of each specific tract. Center, sex, Hoehn-Yahr stage, LEDD and HAMD score were set as nuisance regressors in the first block (model 1) of all regression models whereas PAS score (independent variable) was separately added to the second block (model 2) of the model. We ensured that all models met the assumptions of multiple regression analyses, including normality of the residuals, multicollinearity, and homoscedasticity.

## RESULTS

### Population

After exclusion of patients for dementia (*n* = 14), refusal or contraindication to have an MRI (*n* = 22), unusable 3D-T1w (major motion artefact – *n* = 2), no available DTI (*n* = 1), or unusable DTI after quality control (*n* = 9), 108 participants were included in the present study, 31 with anxiety and 77 without anxiety.

### Demographic and clinical variables

The anxious patients tended to be more frequently female, had a more advanced disease stage, were using a higher LEDD, and more frequently used antidepressants and anxiolytics (Table 1). Logically, PAS total score and sub-scores as well as the HAMD total were higher in the anxious than in the non-anxious group. There was no between-group difference regarding vascular risk factors and cerebral WM changes. The results are detailed in Table 1.

**Table 1:** Demographic and clinical variables: Group comparisons (Parkinson’s disease patients with and without anxiety)

Demographic variables	anxious group (*n* = 31)	non-anxious group (*n* = 77)	OR (CI 95%); *p*
Age (y)	65.91 (±6.30)	64.19 (±8.74)	0.48
Women (*n* = 36)	14 (42.42%)	20 (25.97%)	2.35 (0.98; 5.62); 0.052
Hand dominance (right, *n* = 101)	26 (83.87%)	67 (87.01%)	0.97 (0.28; 3.37); 0.67
Formal education (y)	12.13 (±4.19)	12.60 (±3.60)	0.37
Illness duration (y)	9.29 (±7.45)	8.04 (±4.94)	0.57
**Clinical variables**			
LEDD (mg/day)	944.46 (±511.47)	740.31 (±588.12)	0.028^*^
Antidepressant use (*n* = 17)	12 (38.71%)	4 (5.19%)	11.53 (3.34; 39.81); <0.0001^*^
Anxiolytic use (*n* = 12)	9 (29.03%)	2 (2.60%)	15.34 (3.08; 76.30); <0.0001^*^
MDS-UPDRS part 3 (/132)	38.77 (±13.18)	28.06 (±11.87)	0.73
Hoehn &Yahr stage (0–5) ^§^	2 (1–3)	2 (0–4)	0.010^*^
PAS total (/48)	14.42 (±4.39)	3.66 (±4.35)	<0.0001^*^
*Part A (/20)*	9.32 (±4.35)	2.81 (±2.92)	<0.0001^*^
*Part B (/16)*	2.35 (±2.24)	0.43 (±0.87)	<0.0001^*^
*Part C (/12)*	2.74 (±2.32)	0.43 (±0.84)	<0.0001^*^
HAMD total (/54)	8.87 (±5.36)	4.68 (±3.68)	<0.0001^*^
LARS total	– 21.81 (±9.23)	– 23.91 (±10.35)	0.058
**Vascular risk factor**			
Total vascular risk factor	25 (80.65%)	58 (75.32%)	0.553
- Diabetes	4 (12.90%)	5 (6.49%)	0.275
- Hypertension	11 (35.48%)	18 (23.38%)	0.199
- Hypercholesterolemia	8 (25.81%)	16 (20.78%)	0.570
- Tobacco use	2 (6.45%)	2 (2.60%)	0.324
- Cerebral infarcts	3 (9.68%)	1 (1.30%)	0.070
- Arteriopathy	1 (3.23%)	5 (6.49%)	0.671
Fazekas score^§^
- Periventricular (P)	1 (0–2)	1 (0–2)	0.238
- Deep (D)	1 (0–2)	1 (0–2)	0.799
- Total (P+D)	2 (0–4)	2 (0–4)	0.345

^*^p < 0.05; ^§^CI, confidence interval; HAMD, Hamilton Depression Rating Scale; LARS, Lille Apathy Rating Scale; LEDD, Levodopa Equivalent Daily Dosages; MDS-UPDRS, Movement Disorder Society Unified Parkinson Disease Rating Scale; OR, Odds Ratio; PAS, Parkinson Anxiety Scale.

### MRI analyses

#### Specific tracts creation

After the processing steps, 60 tracts were created bilaterally. Of these, 18 bilateral tracts (i.e. 36 tracts) were included in the analyses: the accumbens-amygdala tract, the accumbens-thalamus tract, the accumbens-insula tract, accumbens-lateral OFC, accumbens-medial OFC, accumbens-right ACC, amygdala-putamen, amygdala-thalamus, amygdala-insula, amygdala- lateral OFC, amygdala-medial OFC, amygdala-right ACC, caudate-left OFC, caudate-medial OFC, caudate-right ACC, putamen-lateral OFC, putamen-medial OFC and thalamus-caudate tract.

### Comparison analyses

In the anxious group, the mean FA value was lower within the left striato-OFC (accumbens-mOFC, caudate-mOFC, putamen-mOFC), left striato-cingulate (accumbens-rACC), left cingulate-limbic (amygdala-rACC) and right striato-thalamic (caudate-thalamus) tracts. It was higher within the right striato-limbic (accumbens-insula) and right striato-thalamic (accumbens-thalamus) tracts compared with the non-anxious group (Table 2).

**Table 2:** Comparison of the mean values of fractional anisotropy (FA) in specific DTI tracts between Parkinson’s disease (PD) patient with and without anxiety

Comparison of FA mean values between anxious and non-anxious PD patients				
Specific tracts	PD with anxiety	PD without anxiety	F	FDR *p*
**Left Tracts**				
L. Accumbens-Amygdala	0.269 (±0.021)	0.274 (±0.025)	2.651	0.05
L. Accumbens-Insula	0.291 (±0.020)	0.296 (±0.023)	1.762	0.16
L. Accumbens-lOFC	0.260 (±0.020)	0.264 (±0.022)	2.667	0.05
**L. Accumbens-mOFC**	0.260 (±0.023)^*^	**0.264 (±0.025)**	**4.180**	**0.012**
**L. Accumbens-rACC**	0.263 (±0.028)^*^	**0.264 (±0.027)**	**3.496**	**0.016**
L. Accumbens-Thalamus	0.307 (±0.025)	0.311 (±0.029)	1.553	0.222
L. Amygdala-Insula	0.273 (±0.023)	0.283 (±0.023)	1.259	0.32
L. Amygdala-lOFC	0.249 (±0.018)	0.256 (±0.019)	1.917	0.13
L. Amygdala-mOFC	0.260 (±0.018)	0.266 (±0.021)	2.262	0.07
L. Amygdala-Putamen	0.274 (±0.021)	0.281 (±0.026)	1.363	0.28
**L. Amygdala-rACC**	0.269 (±0.019)^*^	**0.272 (±0.020)**	**3.955**	**0.012**
L. Amygdala-Thalamus	0.295 (±0.023)	0.303 (±0.024)	1.173	0.35
L. Caudate-lOFC	0.292 (±0.019)	0.296 (±0.022)	2.234	0.07
**L. Caudate-mOFC**	0.310 (±0.023)^*^	**0.312 (±0.023)**	**2.997**	**0.035**
L. Caudate-rACC	0.305 (±0.025)	0.304 (±0.023)	2.508	0.06
L. Putamen-lOFC	0.267 (±0.017)	0.271 (±0.021)	2.237	0.07
**L. Putamen-mOFC**	0.282 (±0.020)^*^	**0.285 (±0.023)**	**3.610**	**0.016**
L. Thalamus-Caudate	0.366 (±0.023)	0.373 (±0.023)	0.568	0.75
**Right Tracts**				
R. Accumbens-Amygdala	0.263 (±0.017)	0.264 (±0.020)	0.660	0.68
**R. Accumbens-Insula**	**0.263 (±0.016)**	0.259 (±0.024)^*^	**3.260**	**0.034**
R. Accumbens-lOFC	0.236 (±0.019)	0.239 (±0.018)	1.504	0.33
R. Accumbens-mOFC	0.237 (±0.022)	0.237 (±0.019)	1.969	0.20
R. Accumbens-rACC	0.238 (±0.023)	0.235 (±0.022)	1.665	0.31
**R. Accumbens-Thalamus**	**0.292 (±0.026)**	0.290 (±0.021)^*^	**3.346**	**0.034**
R. Amygdala-Insula	0.278 (±0.016)	0.284 (±0.024)	0.996	0.53
R. Amygdala-lOFC	0.260 (±0.015)	0.262 (±0.021)	0.804	0.62
R. Amygdala-mOFC	0.261 (±0.014)	0.262 (±0.018)	0.773	0.62
R. Amygdala-Putamen	0.282 (±0.015)	0.285 (±0.024)	1.336	0.37
R. Amygdala-rACC	0.270 (±0.015)	0.270 (±0.018)	1.563	0.33
R. Amygdala-Thalamus	0.319 (±0.012)	0.321 (±0.021)	1.427	0.35
R. Caudate-lOFC	0.274 (±0.019)	0.273 (±0.024)	2.339	0.17
R. Caudate-mOFC	0.263 (±0.023)	0.263 (±0.023)	2.127	0.19
R. Caudate-rACC	0.265 (±0.020)	0.266 (±0.022)	2.061	0.19
R. Putamen-lOFC	0.289 (±0.016)	0.292 (±0.023)	1.076	0.53
R. Putamen-mOFC	0.281 (±0.016)	0.283 (±0.020)	0.987	0.53
**R. Thalamus-Caudate**	0.343 (±0.021)^*^	**0.346 (±0.019)**	**6.091**	**0.0003**

FDR, false discovery rate; mOFC, medial fronto-orbital cortex; lOFC, lateral fronto-orbital cortex; L., left; R., right; rACC, rostral anterior cingulate cortex. Bold, significant difference; ^*^Lower FA between the two groups.

In the anxious group, the mean MD value was higher within the right striato-thalamic (caudate-thalamus) tract and lower within the right striato-limbic (accumbens-insula) tract compared with the non-anxious group (Table 3).

**Table 3:** Comparison of the mean values of mean diffusivity (MD) in specific DTI tracts between Parkinson’s disease (PD) patient with and without anxiety

Comparison of MD mean values between anxious and non-anxious PD patients				
Specific tracts	PD with anxiety	PD without anxiety	F	FDR *p*
**Left Tracts**				
L. Accumbens-Amygdala	0.0010 (±0.0001)	0.0010(±0.0001)	2.215	0.285
L. Accumbens-Insula	0.0010 (±0.0001)	0.0009(±0.0001)	1.102	0.597
L. Accumbens-lOFC	0.0010 (±0.0001)	0.0010(±0.0001)	1.811	0.356
L. Accumbens-mOFC	0.0010 (±0.0001)	0.0010(±0.0001)	1.811	0.356
L. Accumbens-rACC	0.0010 (±0.0001)	0.0010(±0.0001)	1.669	0.356
L. Accumbens-Thalamus	0.0010 (±0.0001)	0.0010(±0.0001)	0.978	0.597
L. Amygdala-Insula	0.0010 (±0.0001)	0.0010(±0.0001)	2.981	0.182
L. Amygdala-lOFC	0.0011 (±0.0001)	0.0011(±0.0001)	1.661	0.356
L. Amygdala-mOFC	0.0011 (±0.0001)	0.0011(±0.0001)	1.473	0.390
L. Amygdala-Putamen	0.0011 (±0.0001)	0.0010(±0.0001)	2.378	0.285
L. Amygdala-rACC	0.0010 (±0.0001)	0.0010(±0.0001)	1.489	0.390
L. Amygdala-Thalamus	0.0010 (±0.0001)	0.0010(±0.0001)	1.410	0.392
L. Caudate-lOFC	0.0009 (±0.0001)	0.0009(±0.0001)	0.901	0.597
L. Caudate-mOFC	0.0009 (±0.0001)	0.0009(±0.0001)	0.625	0.710
L. Caudate-rACC	0.0009 (±0.0001)	0.0009(±0.0001)	0.966	0.597
L. Putamen-lOFC	0.0010 (±0.0001)	0.0010(±0.0001)	0.945	0.597
L. Putamen-mOFC	0.0010 (±0.0001)	0.0010(±0.0001)	0.784	0.657
L. Thalamus-Caudate	0.0009 (±0.0001)	0.0009(±0.0001)	0.646	0.710
**Right Tracts**				
R. Accumbens-Amygdala	0.0011 (±0.0001)	0.0011(±0.0001)	0.588	0.912
**R. Accumbens-Insula**	**0.0010 (±0.0001)**	**0.0011(±0.0001)** ^*^	**3.331**	**0.044**
R. Accumbens-lOFC	0.0011 (±0.0001)	0.0011(±0.0001)	0.706	0.894
R. Accumbens-mOFC	0.0011 (±0.0001)	0.0011(±0.0001)	0.239	0.963
R. Accumbens-rACC	0.0011 (±0.0001)	0.0011(±0.0001)	0.377	0.944
R. Accumbens-Thalamus	0.0010 (±0.0001)	0.0010(±0.0001)	2.375	0.208
R. Amygdala-Insula	0.0011 (±0.0001)	0.0010(±0.0001)	1.125	0.878
R. Amygdala-lOFC	0.0011 (±0.0001)	0.0011(±0.0001)	0.562	0.912
R. Amygdala-mOFC	0.0011 (±0.0001)	0.0011(±0.0001)	0.917	0.881
R. Amygdala-Putamen	0.0011 (±0.0001)	0.0011(±0.0001)	1.062	0.878
R. Amygdala-rACC	0.0010 (±0.0001)	0.0010(±0.0001)	0.780	0.881
R. Amygdala-Thalamus	0.0010 (±0.0001)	0.0010(±0.0001)	0.443	0.944
R. Caudate-lOFC	0.0010 (±0.0001)	0.0010(±0.0001)	2.003	0.259
R. Caudate-mOFC	0.0010 (±0.0001)	0.0010(±0.0001)	1.696	0.389
R. Caudate-rACC	0.0010 (±0.0001)	0.0010(±0.0001)	2.098	0.259
R. Putamen-lOFC	0.0010 (±0.0001)	0.0010(±0.0001)	0.797	0.881
R. Putamen-mOFC	0.0010 (±0.0001)	0.0010(±0.0001)	0.904	0.881
**R. Thalamus-Caudate**	**0.00099 (±0.0001)** ^*^	**0.00098(±0.0001)**	**3.810**	**0.033**

FDR, false discovery rate; mOFC, medial fronto-orbital cortex; lOFC, lateral fronto-orbital cortex; L., left; R., right; rACC, rostral anterior cingulate cortex. Bold, significant difference; ^*^Higher mean MD between the two groups.

### Regression analysis

There was no association between the severity of anxiety and the FA or MD mean values within the tracts studied.

## DISCUSSION

**Fig. 1:**
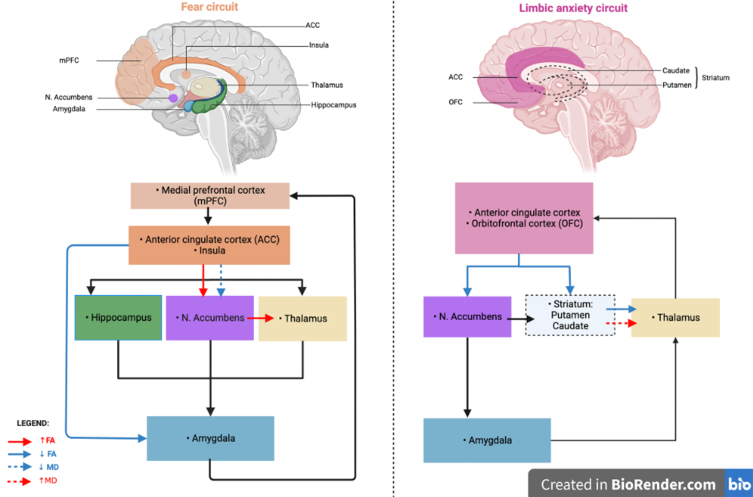
Graphical representation of microstructural alteration in specific tracts of the fear circuit and the limbic anxiety circuit in Parkinson’s disease patients with anxiety. FA, fractional anisotropy; MD, mean diffusivity. Figure created in BioRender.com.

Anxiety is a common non-motor symptom in PD. It is associated with functional and GM changes in neuronal anxiety-related circuits. So far, WM changes related to anxiety were not explored in PD. In this study, PD patients with anxiety had a lower FA within left striato-OFC, left striato-cingulate, left cingulate-limbic and right striato-thalamic tracts as well as a higher FA within right striato-limbic and right striato-thalamic tracts, a higher MD within the right striato-thalamic tract and a lower MD within the right striato-limbic tract compared with the non-anxious group. These results suggest microstructural changes and potential neuronal degeneration in anxiety-related brain circuits. The results are summarized in Fig. 1.

### Microstructural alteration of anxiety-related circuits

FA depicts the summative direction of the diffusion which provides a prominent vector while MD indicates the rate of molecular diffusion [28]. The meaning of FA and MD changes is still debated. FA reduction would refer to the level of disorganization of fibers going into different directions, rather than being organized in a clear pathway, going into the same direction which is the case with high FA. MD would be more specific to axonal changes [29]. Moreover, a reduced FA with increased MD has been associated with degeneration and axonal damage in the WM [28]. Recent studies suggested that DTI parameters would represent axonal and myelin integrity indices. Changes of these parameters could reflect microstructural alterations and axonal or myelin degeneration [10, 11]. In these studies, any change (increase or decrease) of these parameters was associated with microstructural alteration.

We found a reduced FA within striato-OFC, striato-cingulate and striato-thalamic tracts and increased MD within striato-thalamic tract. These tracts are involved in the limbic anxiety circuit [7, 8] (Fig. 1). These results support the hypothesis of neuronal microstructural disorganization of the limbic anxiety circuit in anxious PD patients. This could be associated with the known dopaminergic neuronal degeneration in this neural circuit.

We also found reduced FA within cingulate-limbic tract, increased FA within striato-limbic and striato-thalamic tracts and reduced MD within striato-limbic tract. These tracts are involved in the fear circuit [4–6]. These results also support the involvement of neuronal microstructural disorganization of the fear circuit in anxious PD patients. This increased FA in parts of the fear circuit could reflect a compensatory mechanism for the disorganization in the limbic anxiety circuit. This limbic neuronal disorganization would lead to WM organization within the fear circuit.

In our study, changes in the two involved circuits are opposite, with likely higher FA/lower MD in the fear circuit and lower FA/higher MD in the limbic anxiety circuit. In a recent systematic review, we discussed the substantial overlap between the fear and limbic circuits. The anatomical separation between these circuits may appear artificial but both circuits have already been described and validated independently [4–8]. They can be considered as two parts of a larger limbic circuit. The alteration of several neurotransmission systems (dopamine, serotonin, norepinephrine) due to PD and the resulting dysfunction of the basal ganglia loops could explain the underactivity of the limbic circuit which is involved in cognitive control of emotions [2]. It could promote an hyperactivation of the fear circuit, altering fear processing, as well as an hypoactivation of the limbic circuit, altering the cognitive and behavioral long-term adaptation to fear. The imbalance between these two overlapping circuits could partly explain the high prevalence of anxiety in PD compared with non-PD patients in whom only fear circuit changes are involved in anxiety [12, 13]. These hypotheses are also supported by other independent study groups [3, 30]. In the present study, we suggest that the differences of FA and MD between both circuits support the hypothesis that anxiety in PD could result from an imbalance between the fear and the limbic anxiety circuit [2, 31]. It reveals that anxiety in PD patients is not only associated with GM and functional connectivity changes in anxiety-related circuits but also with microstructural alteration of the WM tracts themselves and structural connectivity changes. Moreover, alterations in WM could also be related to GM changes.

In non-PD anxious patients, DTI abnormalities have been only described within the fear circuit [12, 13]. The results of this study are also consistent with our hypothesis that anxiety in PD patients is a distinct disorder from anxiety in general population. They are also in line with our hypothesis that anxiety in PD is not only a dopaminergic state but also involve extra-striatal structures. So far, only cognitive behavioral therapy was proven effective in reducing anxiety symptoms in PD. CBT was shown to increase functional connectivity between the frontal cortex and striatum, thus strengthening cognitive control over anxiety and restoring the balance between the anxiety and the fear circuit [32]. There is as yet no evidence for the efficacy of any medication in treating these symptoms [33]. A better understanding of the underlying mechanisms of anxiety disorders in PD may facilitate the development of novel therapeutics.

### Strengths and limitations

This is the first study to analyze changes in DTI parameters in PD patients in relation with anxiety. We included a large cohort of patients (*n* = 108) who underwent 3-Tesla MRI scans and standardized clinical evaluation in two sites (Lille and Maastricht). We also compared patients with and without clinically significant anxiety and correlated anxiety symptoms severity to imaging data. However, our study had some limitations. Firstly, the patients were considered to have clinically significant anxiety symptoms according to their score at the PAS. They did not have a formal diagnosis of specific anxiety disorder according to diagnostic criteria (DSM). However, the PAS has demonstrated high sensitivity and specificity for diagnosing anxiety disorders in PD [18]. Secondly, the lack of a healthy control group did not enable us to determine which findings are specific to PD and which findings are not specific and associated with anxiety in general. But the aim of the present study was not to compare anxiety and healthy controls but to compare patients with and without PD-related anxiety. The non-anxious PD patients were considered as the control group. Finally, some structures that may also play a role in anxiety, have not been included in the analyses such as the sub-thalamic nucleus, the ventral tegmental area, the bed nucleus of stria terminalis and other brainstem nuclei (locus coeruleus, raphe nuclei). The 3-Tesla MRI did not allow us to clearly identify these structures. Future studies using 7-Tesla MRI may be necessary to analyze these structures.

### Conclusion

In this study, we found changes in structural connectivity in the two neuronal circuits involved in anxiety in PD patients. These results support our earlier hypothesis that anxiety in PD could result from an imbalance between the fear and the limbic anxiety circuits [2, 31]. Moreover, it reveals that anxiety in PD patients is not only associated with GM and functional changes in anxiety-related circuits but also with microstructural alteration of the WM tracts themselves and structural connectivity changes.

## Data Availability

Data supporting the findings of this study are available from the corresponding author, upon reasonable request.
